# Circulating microRNA-378 levels serve as a novel biomarker for assessing the severity of coronary stenosis in patients with coronary artery disease

**DOI:** 10.1042/BSR20182016

**Published:** 2019-05-17

**Authors:** Hongshi Li, Fei Gao, Xiaowei Wang, Jiahong Wu, Kunze Lu, Minghao Liu, Rongrong Li, Lingling Ding, Rong Wang

**Affiliations:** 1 The Key Laboratory of Cardiovascular Remodeling and Function Research, Chinese Ministry of Education, Chinese National Health Commission and Chinese Academy of Medical Sciences, The State and Shandong Province Joint Key Laboratory of Translational Cardiovascular Medicine, Department of Cardiology, Qilu Hospital of Shandong University, Jinan, Shandong, China; 2 Department of Radiology, Qilu Hospital of Shandong University, Jinan, Shandong, China; 3 The Second Attached Hospital of Fujian Medical University, Fuzhou, Fujian, China; 4 Department of Cardiology, Fuwai Hospital CAMS & PUMC, Beijing, China; 5 Gynecologic Oncology Key Laboratory of Shandong Province, Qilu Hospital of Shandong University, Jinan, Shandong, China; 6 Center for Reproductive Medicine, Provincial Hospital Affiliated to Shandong University, Jinan, Shandong, China

**Keywords:** coronary artery disease, clinical significance, expression, miRNA-378

## Abstract

**Background:** Circulating microRNAs (miRNA) are steady preserved in blood plasma. Multiple evidences have shown that miRNAs play a crucial role in cardiovascular disease including miRNA-378, which has been illustrated to participate in diverse physiological and pathological processes of cardiovascular disease. In the present study, we aim to explore the expression of plasma miRNA-378 and its clinical significance in patients with coronary artery disease (CAD).

**Methods:** MiRNA-378 expression in blood plasma was performed by quantitative real-time PCR (qRT-PCR) in 215 CAD patients and 52 matched controls of healthy populations. Medical information of all patients including the results of coronary angiography (CAG) was acquired through hospital information system (HIS). Spearman’s correlation, binary linear regression, and covariance analysis were used to examine the association between miRNA-378 and relative clinical risk factors. Receiver operating characteristic curve analysis was applied to evaluate the value of miRNA-378 in predicting the disease severity of coronary lesion.

**Results:** Plasma miR-378 expression was significantly down-regulated in CAD patients compared with healthy controls. Relative miR-378 level was shown conversely correlated with Gensini score, which present the severity of coronary artery lesions. Moreover, it is indicated that miR-378 expression can effectively distinguish patients with or without coronary artery stenosis.

**Conclusions:** Plasma miR-378 levels appear to be a promising non-invasive biomarker, but require to be further validated by a large cohort study in future.

## Introduction

Coronary artery disease (CAD), with a high incidence and mortality in the population, is an overwhelming disease that endangers human health throughout the world [1,2]. Coronary artery atherosclerosis is considered the main underlying cause of CAD [3]. However, CAD is diagnosed based on angiography results, which is invasive and technology dependent. Moreover, few of circulating indicators have been reported regarding to the diagnostic value of CAD [4]. Therefore, a circulating indicator for screening coronary lesions is required.

The Gensini scoring system is most widely used in quantifying CAD [5]. The Gensini score not only quantified the degree of stenosis, but also accurately and objectively represent the degree of coronary artery lesions according to different segments of the coronary artery. The calculation method is relatively simple and more suitable for the calculation of large clinical sample size. The higher the score is, the more severe the stenosis will be. Therefore, it proves to be a very practical and effective quantitative evaluation method [6].

MicroRNAs (miRNAs) have been proved to be a group of small non-coding single stranded RNAs of ∼22 nucleotides, which could negatively regulate gene expression by binding to the target mRNAs, degrading mRNA or cleaving target mRNA, repressing mRNA translation [7]. Multiple evidences have shown that miRNAs play a crucial role in cardiovascular disease [8–10]. Importantly, circulating miRNAs are steady preserved in blood plasma because they are either involved in microparticles or bound to proteins, which protected from RNase activity [11]. Therefore, miRNAs in circulation might be usefully served as promising diagnostic biomarkers [12].

Previous research has reported close relationship between miRNA-378 and atherosclerosis [13]. Circulating miRNA-378 was found to remarkably down-regulated in the plasma of CAD patients compared with normal population [14]. Although expression of circulating miRNAs in CAD patients has been reported, the relationship between miR-378 expression and severity of CAD has not been studied. Here, we detected plasma level of miRNA-378 in CAD patients, then investigated the association between miRNA-378 expression and the severity of atherosclerosis lesion.

## Methods and materials

### Ethics statement

The present study was approved by Medical Ethics Committee of Qilu Hospital of Shandong University. Written informed consent was obtained from all of participants before participation.

### Clinical samples

Study participants were enrolled from Qilu Hospital of Shandong University prior to undergoing selective cardiac catheterization. Patients with valvular heart disease, cardiomyopathy, sever infectious disease, tumor, immune disease, and renal dysfunction were excluded. Finally, 267 patients were included in the present study. Patients whose maximal coronary stenosis <50% confirmed by coronary angiograms were included in the control group. Clinical data were collected through hospital information system (HIS). Angiographies were evaluated independently by three cardiologists based on AHA/ACC criteria. Severity of coronary stenosis was demonstrated by Gensini Score according to previous study [15], which was quantified for the degree of luminal narrowing along with a multiplier for specific coronary tree locations for prognostic significance. Each lesion score by weighting summed to give a final Gensini Score.

### Blood sample and miRNA isolation

Venous blood samples (2 ml) were collected before coronary angiography (CAG) in EDTA containing tubes. The sample was centrifuged with 1500 × ***g*** for 15 min at 4°C. 200 μl obtained plasma was subjected to RNA extraction. MiRNA was isolated using miRNeasy Isolation Kit (Qiagen, Hilden, Germany) according to manufacturer’s protocol.

### Measurement of miRNA-378

MiRNAs were reversed transcribed to cDNA using the Reverse Transcription TaqMan MicroRNA Reverse Transcription Kit (Applied Biosystems, Foster City, CA, U.S.A.) according to manufacturer’s instructions. Then, quantitative real-time PCR (qRT-PCR) was performed by Hairpin-it™ microRNA and U6 snRNA Normalization RT-PCR Quantitation Kit (GenePharma, China) in triplicate using Light Cycler (Bio-Rad). U6 was performed as an internal control. The relative expression of miRNA-378 was calculated by methods [14]: *F* = 2^−Δct^, Δct = ct (miRNA-378) – ct (miRNA-U6). CT referred to cycle number inside reactor when the fluorescent signal of sample rose above baseline. MiRNA-378-5p, qPCR Primer Pairs, and U6 primers were synthesized by GenePharma (Shanghai, China). The U6 RT-PCR primers were following: 5′-CGCTTCACGAATTTGCGTGTCAT-3′ and 5′-GCTTCGGCAGCACATATACTAAAAT-3′. MiR-378-5p forward primer: CAAACCTCCTCCTGACTCCAG and reverse primer: TATGCTTGTTCTCGTCTCTGTGTC.

### Statistics

All calculations were performed using SPSS statistical software package. Data from normal distribution were expressed by mean ± SD, and data from abnormal distribution were displayed through median (25th–75th percentile). One-way ANOVA, analysis of covariance (ANCOVA), and nonparametric test adding post LSD were used for comparison of continuous variables among multiple groups. In addition, Student’s *t* test was used for comparison of continuous variables between two groups. Spearman’s correlation was applied for exploring indicated associations. Binary liner regression and multiple linear regression were taken to determine variables that independently contributed to presence of CAD. The receiver operating characteristic (ROC) curve was applied to determine the ability of relative miR-378 levels in predicting coronary stenosis. A difference with *P*<0.05 was considered significant.

## Results

### Demographic characteristics of study patients

The detailed information of patients is demonstrated in Table 1. There were no differences between CAD patients and control group of basic clinical characteristics, including gender, age, blood pressure, heart rate, BMI, Smoking, Drinking, Diabetes, TG, and HDL. Obviously, Gensini Score was significantly higher in CAD group while LDL level was significantly elevated compared with control group. Because control group cannot exclude CAD before coronary arteriography, there was no difference between medication on admission, such as ACEI/ARB, CCB, statins, and nitrates.

**Table 1:** Baseline characteristics of patients with CAD and controls

Variables	All subjects	CAD	Non-CAD	*P*
*N* (male %)	183 (68.5)	153 (71.1)	30 (57.7)	0.06
Age (years)	61±10	61±10	61±12	0.66
SBP (mmHg)	135±20	135±19	136±21	0.68
DBP (mmHg)	76±12	75±12	77±12	0.49
HR (beats/min)	72±12	72±12	73±12	0.19
BMI (kg/m^2^)	22±10	23±10	22±10	0.81
Smoking *n* (%)	124 (46.4)	105 (48.8)	19 (36.5)	0.11
Drinking *n* (%)	81 (30.3)	63 (29.3)	18 (34.6)	0.47
HBP *n* (%)	125 (46.8)	104 (48.4)	21 (40.4)	0.30
Diabetes *n* (%)	71 (26.6)	61 (28.4)	10 (19.2)	0.18
Gensini Score	96±85	111±85	34±50	<0.01
TG (mmol/l)	3.9±1.0	3.9±1.0	3.9±1.0	0.79
HDL (mmol/l)	1.1±0.4	1.1±0.5	1.2±0.3	0.20
LDL (mmol/l)	2.3±0.8	2.4±0.8	2.0±0.5	<0.01
FBG (mmol/l)	5.9±1.9	6.0±2.0	5.6±1.6	0.12
NTproBNP (ng/ml)	402±761	435±835	264±296	0.15
ACEI/ARB *n* (%)	143 (53.6)	119 (55.3)	24 (46.2)	0.32
CCB *n* (%)	93 (34.8)	70 (32.6)	23 (44.2)	0.11
Nitrates *n* (%)	162 (60.1)	135 (62.8)	27 (51.9)	0.15
Statins *n* (%)	26 (99.3)	214 (100.0)	51 (98.1)	0.28
Anti-diabetic agents *n* (%)	71 (26.6)	61 (28.4)	10 (19.2)	0.18

Abbreviations: ACEI/ARB-angiotensin converting enzyme inhibitor/angiotensin receptor blocker; BMI-body mass index; CAD-coronary heart disease; CCB-calcium channel blockers; DBP-diastolic blood pressure; FBG-fast blood glucose; HDL-high-density lipoprotein; HR-heart rate; LDL-low-density lipoprotein; SBP-systolic blood pressure; TC, total cholesterol; TG-total triglyceride.

### Plasma miR-378 levels were down-regulated in CAD patients

**Figure 1:**
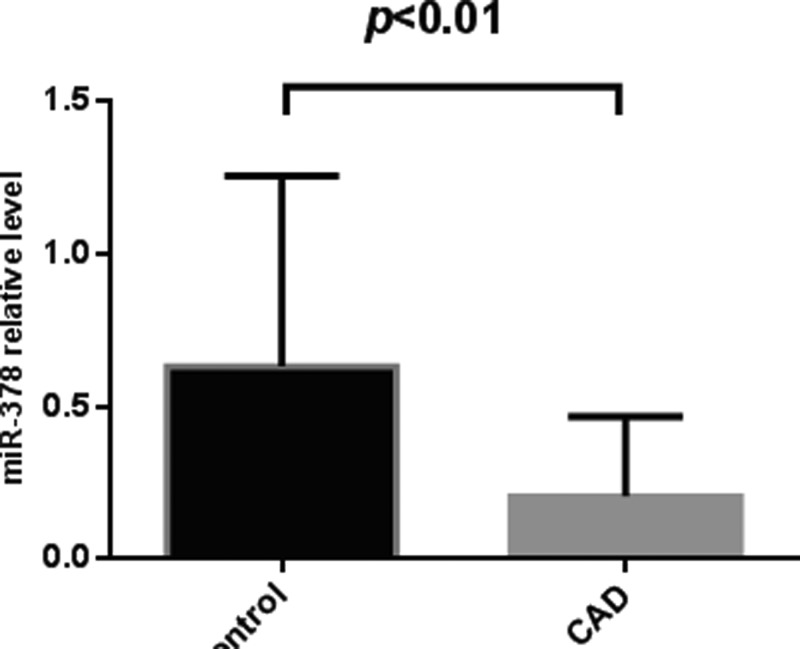
Plasma miR-378 levels between CAD patients and control group Plasma miR-378 levels were detected different between CAD patients (0.6±0.6) and control group (0.2±0.3) by qRT-PCR. The expression levels of miR-378 were normalized to U6. *P* values were calculated using Student’s *t* test. Significant difference was observed about plasma miR-378 levels (*P*<0.01).

We studied the relative levels of miR-378 in plasma of CAD patients and control groups. Student’s *t* tests were used for statistical analysis. Results showed that plasma miR-378 levels were significantly decreased in CAD patients (0.6±0.6) compared with non-CAD controls (0.2±0.3) (Figure 1). In the CAD group the median of the Gensini score is 0.12, and in the control group the median of the Gensini score is 0.35.

### Correlation between relative miR-378 levels and coronary lesion severity

**Figure 2:**
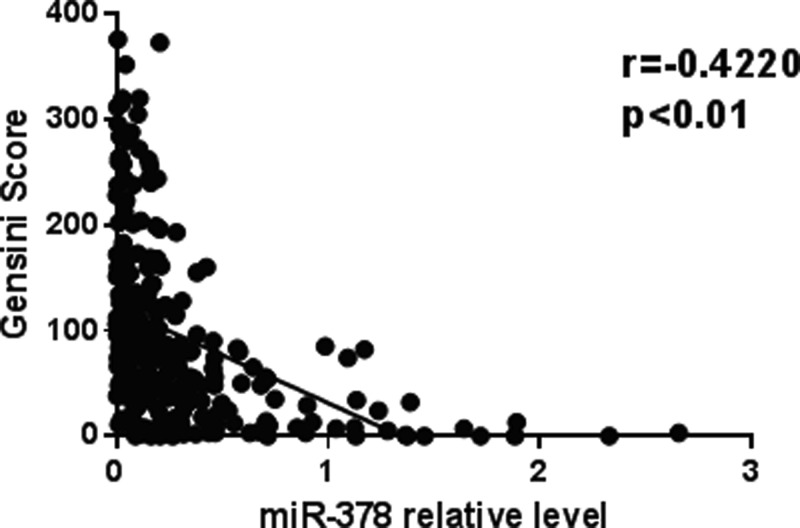
Spearman’s correlation between relative miR-378 levels and Gensini Score The serum relative miR-378 levels were negatively correlated with Gensini Score (*R* = −0.4220; *P* < 0.01).

We further analyzed the association between plasma miR-378 levels and the severity of CAD. The severity of coronary lesion was evaluated with the Gensini Score in the present study (*R* = −0.4220; *P* < 0.01) (Figure 2).

### Plasma miR-378 level was an important risk factor and could predict coronary stenosis

To identify the factors contributed to coronary stenosis, we conducted binary linear regression, with/without CAD to be dependent variable. We found that independent variables (LDL, FBG, and relative miR-378 level) had significant impact on dependent variable (Table 2). After controlling relative covariance, the relative miR-378 level was different among groups (Table 3). To clarify the definite contribute of the risk factors to the miR-378 expression, we conducted multiple liner regression, relative miR-378 levels to be dependent variable. We found that LDL had significant but adverse effect on miR-378 expression, while others showed no significant efficacy in this model (Table 4).

**Table 2:** The risk factor by liner regression in patients with CAD and without CAD

Variables	*P*
LDL	<0.01
FBG	0.02
Relative miR-378 level	<0.01

**Table 3:** The risk factor by covariance analysis with CAD and without CAD

Variables	Sum of squares (type III)	*F*	*P*
LDL	29.07	1.39	0.36
FBG	31.48	1.00	0.51
Relative miR-378 level	38.52	2.03	0.04

**Table 4:** Relative miR-378 level and the risk factors are compared with multiple linear regression

	β	*t*	*P*
LDL	−0.138	−2.266	0.02

**Figure 3:**
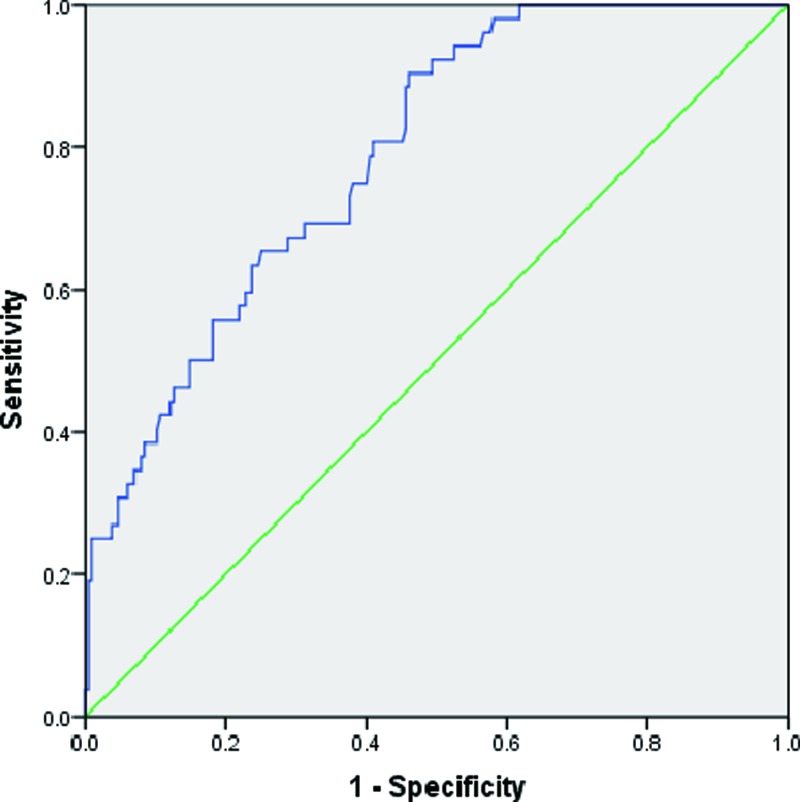
AUC of ROC was used to study the predictive value of CAD patients The AUC is 0.789 (*P*<0.01).

Then, ROC curve was applied to evaluate relative miR-378 levels in predicting coronary stenosis. The area under curve (AUC) is 0.789 (*P*<0.01) (Figure 3). MiR-378 expression could be used to predict coronary lesions (Table 5). These results indicated that miR-378 had an important prediction value for diagnosis of CAD and decreased plasma miR-378 levels were associated with increasing of severity of CAD.

**Table 5:** Evaluation of relative miR-378 level by ROC curve in diagnosis of CAD

	AUC	95%CI	*P*
Relative miR-378 level	0.789	0.728–0.851	<0.01

## Discussion

MiRNAs had been demonstrated highly conserved and played much vital roles in cardiovascular disease [8–10]. Recent studies had also shown that circulating miRNAs could act as novel biomarkers for diagnosis of CAD [12]. CAD currently was leading cause of mortality around the world, mainly caused by atherosclerosis. Therefore, early diagnosis was key to treatment of CAD. However, imaging technology was considered gold standard in routine clinical practice. Therefore, a potential biomarker for evaluating the presence and the severity of CAD was needed.

Circulating miRNA-378 was found to remarkably down-regulated in the plasma of CAD patients compared with normal population [16]. MiRNA-378 had been illustrated to participate in diverse physiological and pathological processes. It was reported that expression of miR-378 enhanced cell survival, reduced caspase-3 activity, and promoted angiogenesis [17–19]. Previous studies also demonstrated that miR-378 was as a regulator of cardiomyocyte hypertrophy, which exerted its activity by suppressing MAPK and Ras signaling pathway [20,21]. Kim et al. identified that miR-378 could be as a connective tissue growth factor (Ctgf) regulator in electrical stimulation cardiac stem cells. Ctgf was responsible for stem cell survival and adhesion [22]. Templin et al. reported that miR-378 regulated angiogenic capacity of CD34+ progenitor cells in myocardial infarction [23]. BMP4 was confirmed to be a direct target of miRNA-378 [24]. During the development of atherosclerosis, BMP4 produced in endothelial cells, which then leads to ICAM-1 induction and monocyte binding [25]. BMP4 accelerate the progression of atherosclerosis by induced macrophage foam cell formation through BMPR-2/Smad1/5/8 signaling [26]. Matkovich et al. predicted miRNA-378 should regulate miR-155 [27]; miR-155 have been suggested in high expression levels in pro-inflammatory macrophages in atherosclerotic plaques induced by oxidized LDL [28]. CD14+ monocytic cells have also shown increased miR-155 expression in patients with CAD compared with healthy subjects [28]. Those results indicated that miR-378 might exert a beneficial effect on CAD.

In the present study, we evaluated the relative level of plasma miR-378 in CAD patients whose angiography demonstrated at least one major epicardial artery with >50% stenosis and control group without CAD, which indicated that miR-378 might have a potential impact on predicting coronary lesions for CAD. Plasma miR-378 expression was significantly down-regulated in CAD patients than that of control group. Our result was consistent with Weber’s report [14]. Relative miR-378 level was shown conversely correlated with Gensini Score, which presented the severity of coronary artery lesions. Previous studies had shown that LDL and FBG are risk factors of CAD [29,30]. The same results were confirmed by our study. Moreover, our study revealed that miR-378 level, which was associated with LDL, was also a risk factor of CAD, and had significant difference with others. MiR-378 could effectively distinguish patients with or without coronary artery stenosis, which suggested that miRNA-378 could be used as a biological indicator for estimating coronary lesions. And further studies indicated that the association of miR-378 and CAD directly were required.

There were still several limitations in present study. Given significant cost involved in RT-PCR studies, clinical sample should be increased to enhance the power of statistical analysis. Secondly, we did not illustrate direct relationship in the present study between miR-378, LDL and CAD. Mechanistic studies were needed to confirm its potential as a biomarker.

In conclusion, we found that relative miR-378 levels were significantly lower in CAD patients compared with non-CAD subjects, which had significantly relationship with severity of coronary stenosis. Plasma miR-378 expression could provide further evidence for clinical practice in the diagnosis of CAD.

## Abbreviations

AUC: area under curve
CAD: coronary artery disease
Ctgf: connective tissue growth factor
miRNA: microRNA
qRT-PCR: quantitative real-time PCR
ROC: receiver operating characteristic
